# Unraveling the Complex Physiological, Biochemical, and Transcriptomic Responses of Pea Sprouts to Salinity Stress

**DOI:** 10.3390/genes16091043

**Published:** 2025-09-03

**Authors:** Xiaoyu Xie, Liqing Zhan, Xiuxiu Su, Tingqin Wang

**Affiliations:** College of Coastal Agricultural Sciences, Guangdong Ocean University, Zhanjiang 524088, China

**Keywords:** *Pisum sativum*, salinity, stress response

## Abstract

**Background**: The escalating global salinization poses a significant threat to agricultural productivity, necessitating a thorough understanding of plant responses to high salinity. Pea sprouts (*Pisum sativum*), a nutrient-rich crop increasingly cultivated in salinized regions, serve as an ideal model for such investigations due to their rapid growth cycle and documented sensitivity to ionic stress. **Methods**: In order to understand the response of pea sprouts in physiological regulation, redox-metabolic adjustments, and transcriptome reprogramming under salt stress, we investigated the effects of high salt concentrations on the ascorbic acid–glutathione cycle, endogenous hormone levels, metabolite profiles, and gene expression patterns in it. **Results**: Our findings reveal early-phase antioxidant/hormonal adjustments, mid-phase metabolic shifts, and late-phase transcriptomic reprogramming of pea sprouts under salt conditions. In addition, a biphasic response in the ascorbic acid cycle was found, with initial increases in enzyme activities followed by a decline, suggesting a temporary enhancement of antioxidant defenses. Hormonal profiling indicated a significant increase in abscisic acid (ABA) and jasmonic acid (JA), paralleled by a decrease in indole acetic acid (IAA) and dihydrozeatin (DZ), underscoring the role of hormonal regulation in stress adaptation. Metabolomic analysis uncovered salt-induced perturbations in sugars, amino acids, and organic acids, reflecting the metabolic reconfiguration necessary for osmotic adjustment and energy reallocation. Transcriptomic analysis identified 6219 differentially expressed genes (DEGs), with a focus on photosynthesis, hormone signaling, and stress-responsive pathways, providing insights into the molecular underpinnings of salt tolerance. **Conclusions**: This comprehensive study offers novel insights into the complex mechanisms employed by pea sprouts to combat salinity stress, contributing to the understanding of plant salt tolerance and potentially guiding the development of salt-resistant crop varieties.

## 1. Introduction

Sprouting vegetables, commonly known as “sprouts,” encompass a diverse array of beans, cereals, and vegetables, with their youthful stems, sprouts, and hypocotyls serving as primary edible components [1]. Beyond their rich nutrient content, these vegetables offer a crisp texture [2] and have a longstanding history of popularity among consumers [3]. Notably, pea sprouts (*Pisum sativum*) are abundant in essential nutrients such as vitamin C, carotene, potassium, vitamin B, and various essential amino acids crucial for human health [2], showcasing high nutritional value and significant potential for enhanced production [1]. Characterized by a soft texture, smoothness, and delightful taste, pea sprouts excel in their color, aroma, and flavor [2], while also aiding in appetite improvement, promoting gastrointestinal motility, and boosting the body’s resilience [3]. Positioned as a novel, healthful, and verdant vegetable option, pea sprouts present promising market opportunities and substantial developmental possibilities.

In the backdrop of global warming and climate fluctuations, numerous soils worldwide have become salinized [3], especially in arid regions where saltwater irrigation is prevalent. Consequently, post-drought salt damage has emerged as a significant environmental challenge impeding plant development [3]. As environmental salt concentrations escalate, the focus on plant responses to salt stress intensifies, underscoring a growing interest in their stress-resistant mechanisms [3]. Beyond nutritional density, the high sensitivity to ionic stress of pea sprouts offers it a unique model for stress chronobiology [1]. The rapid germination of pea sprouts (3–5 days) permits hourly tracking of molecular responses, unlike slower-growing crops. This addresses the ‘snapshot’ limitation plaguing salt-stress studies in perennial plants [4]. While existing research primarily concentrates on salt-tolerant crops like rice in the Gramineae family, cruciferous vegetables, and peanuts in the Leguminosae family [2], investigations into the salt stress responses of pea sprouts remain relatively scarce. Previous studies have indicated that a certain degree of salt stress levels can enhance the nutritional quality and growth rate of pea sprouts; however, excessive salt stress may impede sprout growth, impacting yield and quality [1,3]. Nonetheless, the precise physiological and biochemical mechanisms governing the response of pea sprouts to elevated salt stress remain inadequately understood.

In the face of salt stress, plants release a substantial quantity of reactive oxygen species (ROS), which have the potential to harm membrane lipids and induce oxidative stress, thereby detrimentally impacting plant health. This oxidative stress arises from the inability of membranes to fully shield against the deleterious effects of these free radicals, culminating in cellular impairment [5,6]. The ascorbate–glutathione (AsA-GSH) cycle is a central ROS-scavenging hub under salinity [6], synergistic interactions with other antioxidants—including superoxide dismutase (SOD), catalase (CAT), and peroxidases—are critical for full oxidative stress mitigation [5]. While SOD and CAT contribute to ROS scavenging, the AsA-GSH cycle represents the core redox buffer system under salinity due to its ability to regenerate antioxidants and interface with stress signaling networks [6]. Specifically, it detoxifies H_2_O_2_ in chloroplasts and mitochondria—organelles highly vulnerable to salt-induced ionic imbalance—and its components (e.g., AsA, GSH) serve as co-factors for ABA biosynthesis enzymes [7]. This positions the AsA-GSH cycle not merely as an antioxidant pathway but as a strategic nexus coordinating redox homeostasis and stress signaling. The AsA-GSH system comprises non-enzymatic antioxidants such as ascorbate (AsA) and glutathione (GSH), in conjunction with vital enzymatic constituents like monodehydroascorbate reductase (MDHAR) and dehydroascorbate reductase (DHAR), which play pivotal roles in the plant’s antioxidant defense mechanisms [7,8]. Furthermore, the conversion of the oxidized form of glutathione (GSSG) back to GSH is facilitated by the action of glutathione reductase (GR), thereby preserving the cellular redox equilibrium [9]. Studies have underscored the indispensable nature of the AsA-GSH cycle in the response of plant seedlings to salt stress, offering crucial insights into their salt tolerance attributes [7,8]. Extensive research has elucidated the pivotal role of this cycle in shielding plants from oxidative harm triggered by environmental stresses [6].

Another significant mechanism through which plants resist external stressors is the secretion of endogenous hormones. Abscisic acid (ABA) plays a crucial role in aiding plants in adapting to challenging conditions. Particularly under salt stress, ABA levels witness a substantial surge, predominantly as a response from plant leaves [9]. Other hormones, such as auxin (IAA) and gibberellic acid (GA), also contribute to plant growth and development amid stress conditions. GA collaborates with other hormones to facilitate standard maturation processes in plants confronting salt stress [5,9]. The equilibrium between ABA and zeatin (ZR) holds particular significance in regulating stomatal aperture, a crucial adaptation to environmental stresses [5,9]. Previous investigations have emphasized that the ratio of IAA, ABA, and ZR plays a critical role in determining a plant’s efficacy in withstanding environmental stresses. The intricate interplay between growth-promoting hormones like IAA and GA and the growth-inhibiting hormone ABA elucidates how hormonal adjustments aid plants in managing stress [6,9]. Nevertheless, the precise mechanisms of hormonal regulation in pea sprouts under salt stress remain ambiguous, necessitating further exploration.

The study of sugars, organic acids, amino acids, and coenzymes in pea sprouts under salt stress is driven by their interconnected roles in orchestrating metabolic adjustments, maintaining cellular homeostasis, and mitigating stress-induced damage. Sugars, such as sucrose and phosphorylated intermediates like sedoheptulose-7-phosphate (S7P) and ribulose-1,5-bisphosphate (RuBP), are critical for osmotic regulation and signaling. During salinity, sucrose accumulation stabilizes cellular osmotic potential, while shifts in Calvin cycle intermediates reflect a redirection of carbon resources from growth to protective mechanisms, a response well-documented in salt-stressed legumes [10]. Organic acids, including citrate, malate, and phosphoenolpyruvate (PEP), serve as linchpins in energy metabolism and pH regulation [11,12,13,14]. Citrate modulates ATP production within the TCA cycle, often decreasing under salt stress due to impaired respiratory efficiency, while the malate-PEP shuttle sustains cytosolic pH and redox balance amid ion toxicity [15,16]. Amino acids, notably serine, contribute to stress adaptation by supporting protein synthesis and acting as precursors for antioxidants like glutathione, which counters oxidative damage [17]. Coenzymes such as NADP+, FAD, and ADP act as metabolic indicators and facilitators; NADP+ fuels the oxidative pentose phosphate pathway to supply NADPH for antioxidant systems, while FAD supports mitochondrial electron transport, both of which are disrupted under salinity [17]. These metabolites do not operate independently—sugar levels influence organic acid production, and coenzymes regulate the enzymatic interplay among them, forming a dynamic network that dictates stress resilience. Consequently, examining these compounds collectively unveils a holistic picture of metabolic trade-offs and adaptive strategies in pea sprouts, resonating with contemporary plant metabolomics approaches that leverage multi-metabolite analyses to decode complex stress responses [18].

Transcriptomics investigates disparities and regulatory mechanisms of gene expression at the genomic level. In plants, transcriptome analysis has emerged as a pivotal approach for examining plant growth, development, stress responses, metabolic regulations, and offering insights into the dynamic shifts in plant gene expression [19]. Although studies have elucidated salt responses in mature pea plants [20], no studies have resolved the genome-wide reprogramming in pea sprouts during early salinity exposure—a phase where rapid metabolic decisions dictate survival. The AsA-GSH cycle interfaces with ABA signaling through glutathione (GSH)-mediated activation of ABA biosynthetic enzymes (e.g., AO_3_), while ABA reciprocally induces AsA-GSH gene expression via ABRE cis-elements [8,21]. This positions ABA as the central transducer that converts early redox changes into transcriptomic reprogramming [21]. Consequently, the tracking of ABA dynamics as the mechanistic linchpin could bridge targeted biochemistry (AsA-GSH, metabolites) and genome-wide responses.

To address the challenges posed by salt stress in pea sprout cultivation and enhance its yield and quality, this study delved into the physiological, biochemical, and molecular responses of pea sprouts under high salt stress. To capture the systemic nature of salt adaptation, four interconnected tiers were tracked: (i) ROS scavenging via the AsA-GSH cycle; (ii) hormonal cross-talk fine-tuning growth arrest and defense; (iii) metabolite flux toward osmoprotection; and (iv) genome-wide transcriptional regulation. This hierarchical design bridges biochemical plasticity with gene regulatory networks, offering a holistic view of salinity resilience. We hypothesize that salt stress triggers a phased defense response in pea sprouts, where early ROS-ABA signaling activates transient antioxidant buffering via the AsA-GSH cycle, followed by metabolic reallocation toward osmotic adjustment, and late-stage transcriptomic reprogramming to consolidate stress tolerance. This research endeavor aims to advance the optimized utilization of pea sprouts in agricultural practices and concurrently furnish valuable insights for the exploitation and development of salt-tolerant crop resources.

## 2. Materials and Methods

### 2.1. Materials and Experimental Design

Pea seeds (*Pisum sativum* cv. Zhongwan No. 6) were carefully selected based on criteria including full grains, uniform size, absence of external injuries, diseases, and insect pests. The seeds were washed thoroughly with clean water 2 to 3 times, soaked in 700 mL of room temperature water for 19 h, and subsequently spread evenly in the grid of a seedling tray, with 80 g of seeds per tray. A twice-daily water spray regimen was implemented in the morning and afternoon. Upon reaching a root length of 1 cm, water was added beneath the seedling tray, the lid was removed, and the seedlings were cultivated under scattered room temperature light. Seedlings were cultivated under 100 μmol m^−2^ s^−1^ PAR (12-h photoperiod, white LEDs), 25/20 °C (day/night), and 70% relative humidity. When the seedlings reached a height of 3 cm, they were subjected to a 200 mmol/L saline treatment, with clean water serving as the control. Salt treatment was applied via root-zone irrigation (200 mL per tray daily at 9:00 AM), avoiding foliar contact. Controls received equivalent deionized water. This method ensures uniform rhizosphere salinity without osmotic shock [22]. Sampling was conducted daily from day 0 to day 4 after the treatment, with 3 replicates for each treatment.

### 2.2. Ascorbic Acid–Glutathione Cycle

One gram of sprout seedling sample was weighed, washed, and placed in a pre-cooled mortar. Subsequently, 5 mL of 0.05 mol/L pre-cooled phosphate buffer (pH 7.8) was added three times to create a homogenate on an ice bath. The resulting mixture was transferred to a centrifuge tube and centrifuged at 4 °C, 12,000× *g* for 20 min to obtain the crude extract.

Following Nakano’s method [23], ascorbate peroxidase (APX) activity was determined by dissolving 0.10 mL of the enzyme solution in 2.6 mL of EDTA-Na_2_, adding 0.15 mL of AsA, and subsequently introducing 0.15 mL of H_2_O_2_ into the solution. The mixture was then kept at 20 °C, and the OD_290_ value was measured every 30 s four times. Absorbance was measured using a Shimadzu UV-2600i spectrophotometer (Kyoto, Japan) with 1 cm pathlength quartz cuvettes, 2 nm slit width, and medium scan speed. One unit of APX activity was defined as an absorbance change of 0.01 unit min^−1^, and the APX activity was expressed as U g^−1^ FW.

MDHAR activity was assessed based on the method outlined by Ma and Cheng [24]. The supernatant was mixed with 2.9 mL of 50 mmol L^−1^ Hepes-NaOH (pH 7.6) containing NADH, AsA, and ascorbate oxidase, and the mixture was scanned at a wavelength of 340 nm at 30 s intervals. Enzyme activity was defined as 0.01 of the change in OD_340_ value within 30 s, equating to one enzyme activity unit (U). DHAR activity was determined using a similar approach as MDHAR, with 2.9 mL of 100 mmol L^−1^ Hepes-NaOH (pH 7.0) containing GSH and DHA, and enzyme activity was calculated based on the change of 0.01 of the OD_265_ value within 30 s, representing one enzyme activity unit (U).

The Ascorbic Acid (AsA) content was quantified utilizing the standard curve method [25]. Initially, 6 mL of 5% trichloroacetic acid (obtained from Sinopharm Chemical Reagent Co., Ltd. Shanghai, China) was added to 1.0 g of the sample, which was then ground in an ice bath at 4 °C and centrifuged at 20,000 r/min for 15 min. The resulting supernatant was utilized for AsA content assessment. For the analysis, 1.0 mL of the sample extract was transferred to a test tube, followed by the addition of 1.0 mL of 5% TCA and 1.0 mL of ethanol (from Sinopharm Chemical Reagent Co., Ltd.), with the mixture thoroughly shaken. Subsequently, 0.5 mL of 0.4% H_3_PO_4_-ethanol, 1.0 mL of 0.5% 2,2′-bipyridine-ethanol, and 0.5 mL of 0.03% FeCl_3_-ethanol were sequentially added, reaching a total volume of 5.0 mL. The solution was then incubated at 30 °C for 90 min, following which the optical density (OD) at 534 nm was measured using a spectrophotometer to determine the AsA content based on the calibration curve established with known concentrations.

The Glutathione (GSH) content was determined via spectrophotometry [26]. A leaf sample weighing 1.0 g was taken, and 5 mL of 5% metaphosphoric acid solution (from Guangdong Guanghua Technology Co., Ltd. Guangzhou, China) was added. The mixture was ground at 4 °C and subsequently centrifuged at 20,000 r/min for 15 min, with the resulting supernatant employed for analysis and stored at 5 °C. To initiate the assay, a reduced glutathione standard solution was prepared, and a standard curve was constructed. The spectrophotometer was calibrated using a blank control tube (ODc) and a sample tube (ODs). Following a 10 min incubation at 30 °C, the absorbance value was measured at a wavelength of 412 nm. After the color reaction, the absorbance of the sample tube mixture (ODs) and the absorbance of the blank control tube reaction mixture (ODc) were recorded. The variance in absorbance values was utilized to ascertain the corresponding quantity of reduced glutathione from the standard curve, facilitating the calculation of the reduced glutathione content in μmol/g.

### 2.3. Determination of Endogenous Hormone Content

A total of 50 mg samples were extracted from cryopreserved biomaterials and mixed with appropriate internal standards after pulverization. The mixture was concentrated at a ratio of 15%:4:1 (*v*:*v*:*v*) and subsequently redissolved in a 100 μL solution of 80% methanol–water to complete the extraction process. Following filtration, the sample was transferred to an injection bottle for efficient LC-MS/MS analysis. Qualitative and quantitative analysis of hormones was performed using established methods that are sensitive and specific for detecting low concentrations of various endogenous hormones in plant tissues [27].

### 2.4. Targeted Metabolome Analysis

The metabolites are strategically positioned at metabolic branch points: sugars (Sedoheptulose-7-phosphate (S7P), ribulose-1,5-bisphosphate (RuBP), erythrose-4-phosphate (E4P) for osmolyte synthesis; serine for glutathione precursors; organic acids (PGA, PEP) for energy shunting; and co-factors (FAD, NADP) as redox sensors. This focused design maximizes resolution of critical pathway fluxes under salt stress [28]. Metabolites were quantified by a revised methodology that follows established protocols for plant stress metabolomics [29,30]. Metabolite extraction was performed using 100 mg of flash-frozen pea sprout tissue (3 cm height, Day 4 post-treatment) homogenized in 900 μL of ice-cold 50% methanol: water (*v*/*v*, HPLC grade) with 0.1% formic acid. Homogenates were vortexed (1 min), sonicated (15 min, 4°C), and centrifuged (15,000× *g*, 15 min, 4 °C). Supernatants were filtered through 0.22-μm PTFE membranes. Chromatographic separation was employed using a Shimadzu Nexera UHPLC system (LabSolutions LCMS 5.99) with an ACQUITY UPLC^®^ HSS T3 column (2.1 × 100 mm, 1.8 μm; Waters, Milford, MA, USA). Mass spectrometric detection utilized a Sciex QTRAP^®^ 6500+ system (Analyst 1.6.3) with electrospray ionization (ESI) in positive/negative switching mode. Optimized MRM transitions for all 11 metabolites were validated using authentic standards (Sigma-Aldrich, St. Louis, MO, USA). Quantification relied on external calibration curves (0.1–100 μg/mL, R^2^ > 0.99). Data processing used Sciex OS Software v3.0, with peak integration manually verified.

### 2.5. Transcriptomics Analysis

After the leaves were ground in liquid nitrogen, RNA was extracted, and the residual genomic DNA was digested. The quantity and quality of total RNA were determined by spectrophotometry and electrophoresis, followed by cDNA encoding, culminating in efficient transcriptome sequencing using the second-generation Illumina NovaSeq platform (Illumina Inc., San Diego, CA, USA). Initial sequencing results in acquiring raw reads. Subsequently, reads with low quality, adapter contamination, and high N base content were eliminated, yielding clean reads. Alignment of clean reads to the reference genome facilitated the prediction of new transcripts, ultimately forming a comprehensive reference sequence. Transcript expression levels were assessed using the FPKM method. Expression differences between experimental groups were determined based on an FDR (false discovery rate) ≤ 0.05 and |log_2_FC| ≥ 1, with functional characteristics of DEGs extensively examined through GO and KEGG methodologies.

### 2.6. Statistical Analysis

Data analysis and statistical procedures in this study were executed utilizing WPS 2019 and SPSS 26.0 software to ensure data accuracy and reliability. Results were presented as mean values with standard deviations. Analysis of variance, followed by post hoc Fisher Least Significant Difference test (FLSD), was employed for comparing treatment means. Furthermore, Origin 2021 software facilitated graphical representation, enabling the visual elucidation of data distinctions.

## 3. Results

### 3.1. Effect of Salt Stress on the Ascorbic Acid Cycle in Pea Sprouts

**Figure 1:**
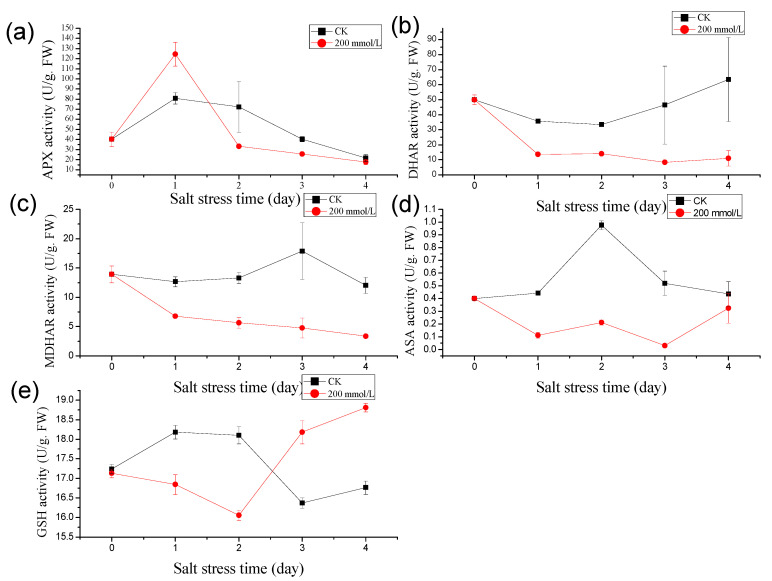
Effects of salt stress (200 mmol/L NaCl, Days 0–4) on antioxidant activity in pea sprouts: (**a**) Ascorbate peroxidase (APX) activity; (**b**) Dehydroascorbate reductase (DHAR) activity; (**c**) Monodehydroascorbate reductase (MDHAR) activity; (**d**) Ascorbic acid (AsA) content; (**e**) Glutathione (GSH) content. Data = mean ± SD (*n* = 3).

In response to salt stress, the activity of ascorbate peroxidase (APX) in pea sprouts exhibited an initial increase followed by a subsequent decrease (Figure 1a). After one day of salt stress treatment, the plant’s APX enzyme activity notably surpassed that of the control, registering at 1.55 times higher than the control level. Over the subsequent 2, 3, and 4 days of salt stress exposure, the APX enzyme activities in pea sprouts were recorded at 46.16%, 63.27%, and 81.49% of the control, respectively, with a diminishing decline trend observed over time. This pattern indicates a transient significant increase in APX enzyme activity under salt stress, gradually declining with prolonged exposure.

Similarly, under salt treatment, the dehydroascorbate reductase (DHAR) activity in pea sprouts depicted an initial decline, followed by an increase (Figure 1b). Following one day of salt treatment, the DHAR activity in the treatment group notably lagged behind the control. The DHAR enzyme activities recorded over the 1 to 4 days post-salt treatment were 38.39%, 41.90%, 18.07%, and 17.49% of the control, respectively, underscoring a substantial decrease in DHAR activity under salt stress.

Contrasting with the control group, the MDHAR activity in pea sprouts exhibited a pronounced decreasing trend under salt treatment. The MDHAR activities recorded after 1 to 4 days of salt treatment were 53.55%, 42.39%, 26.66%, and 27.97% of those in the control group, respectively (Figure 1c).

Moreover, the ascorbic acid (AsA) content in pea sprouts under salt stress displayed an initial rise followed by a decline in comparison to the control group (Figure 1d). Unlike the control, the AsA content significantly decreased after 1 to 3 days of salt treatment, reaching levels of 25.22%, 21.73%, and 6.01% of the control, respectively. After 4 days of salt treatment, the AsA content rebounded to 74.13% of the control. Additionally, under high salt stress conditions, the glutathione (GSH) content in pea sprouts manifested an initial decrease, followed by an increase. The GSH content after 1 to 4 days of salt treatment was measured at 92.63%, 88.69%, 111.06%, and 112.32% of the control, respectively (Figure 1e).

### 3.2. Effect of Salt Stress on Endogenous Hormone Content in Pea Sprout Leaves

**Figure 2:**
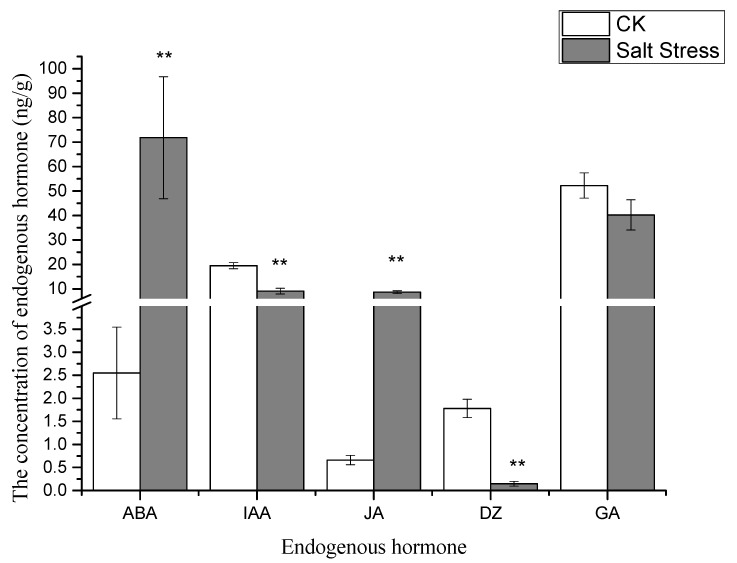
Endogenous hormone profiles in pea sprout leaves under salt stress: abscisic acid (ABA), jasmonic acid (JA), indole acetic acid (IAA), and dihydrozeatin (DZ). Data = mean ± SD (*n* = 3). Asterisks (**) indicate significant difference (*p* < 0.01, *t*-test).

The levels of abscisic acid (ABA) and jasmonic acid (JA) in pea sprouts exhibited significant increases under salt stress compared to the control group, while the concentrations of indole acetic acid (IAA) and dihydrozeatin (DZ) were notably lower than those in the control group (Figure 2).

### 3.3. Effects of Salt Stress on Metabolites of Pea Sprout Leaves

Metabolome analysis identified a total of 11 distinct metabolites. The predominant differentially expressed metabolites encompass sugars, amino acids, and organic acids. Specifically, the differentially expressed metabolites include four types of carbohydrates—RuBP, S7P, E4P, and Sucrose; one type of amino acid—Serine; and three types of organic acids—PGA, PEP, and Malate, alongside metabolites like ADP, FAD, and NADP. The concentrations of sugars, amino acids, and organic acids exhibited a declining trend under salt stress, with notable differences observed in the metabolite levels between the experimental and control groups.

**Figure 3:**
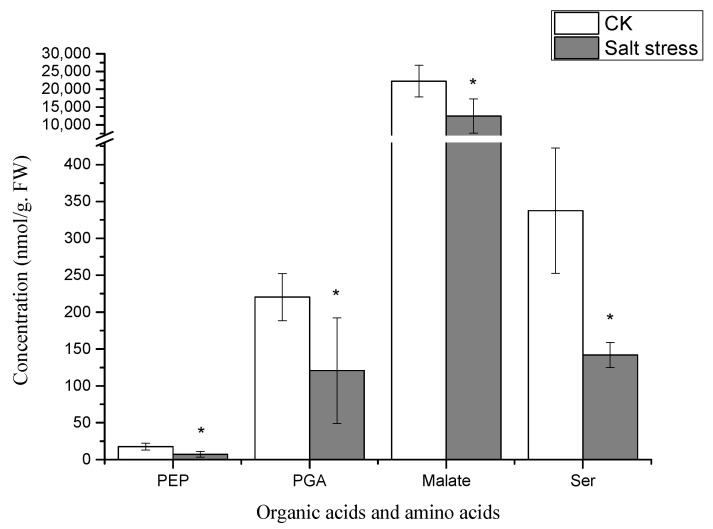
Relative concentrations of organic acids and amino acids in salt-stressed pea sprouts: phosphoenolpyruvate (PEP), phosphoglyceric acid (PGA), malate, and serine. Data = mean ± SD (*n* = 3). Asterisks (*) indicate significant reductions (*p* < 0.05, *t*-test).

**Figure 4:**
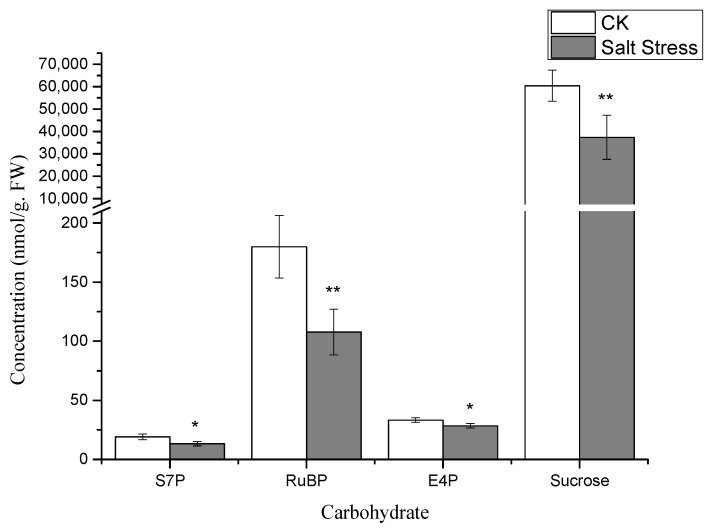
The concentrations of carbohydrate metabolites in pea sprouts under salt stress: sedoheptulose-7-phosphate (S7P), ribulose-1,5-bisphosphate (RuBP), erythrose-4-phosphate (E4P), and sucrose. Data = mean ± SD (*n* = 3). Asterisks (*) indicate significant reductions (*, *p* < 0.05; **, *p* < 0.01).

**Figure 5:**
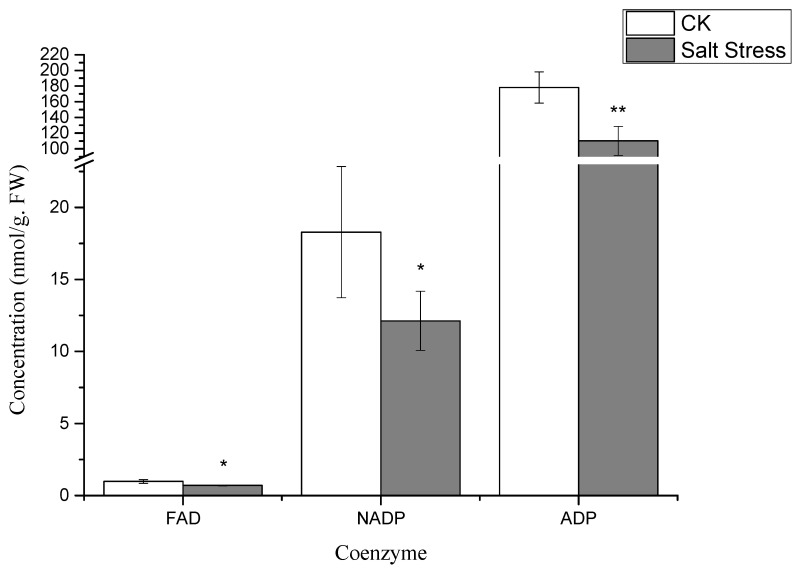
Changes in coenzyme concentrations under salt stress: flavin adenine dinucleotide (FAD), nicotinamide adenine dinucleotide phosphate (NADP), and adenosine diphosphate (ADP). Data = mean ± SD (*n* = 3). Asterisks (*) mark significant differences (*, *p* < 0.05; **, *p* < 0.01).

Notably, under high salt stress conditions, the serine content in pea sprouts was recorded at 50% of that in the control group (*p* < 0.05) (Figure 3). The organic acid content in pea sprout leaves demonstrated significant alterations in response to salt stress, with the relative contents of PGA, PEP, and Malate notably lower than those in the control group (*p* < 0.05) (Figure 3). Furthermore, the relative contents of S7P, RuBP, E4P, and sucrose under high salt stress also exhibited significant reductions in comparison to the control group (Figure 4). Additionally, the levels of FAD, NADP, and ADP in pea sprout leaves were significantly diminished under salt stress conditions (Figure 5).

### 3.4. Analysis of the Transcriptome of Pea Sprouts Under Salt Stress

The clean reads obtained in this study showcased a Q30 base percentage of 93.49%, indicating the accuracy of the sequencing outcomes for subsequent analyses. Furthermore, alignment of the clean reads with the reference genome of pea sprouts revealed an alignment efficiency ranging between 93.20% and 95.20%. Compared with the control group, 6219 differentially expressed genes were obtained in the cDNA library of plant leaves after salt treatment, including 2588 upregulated genes and 3631 downregulated genes.

**Figure 6:**
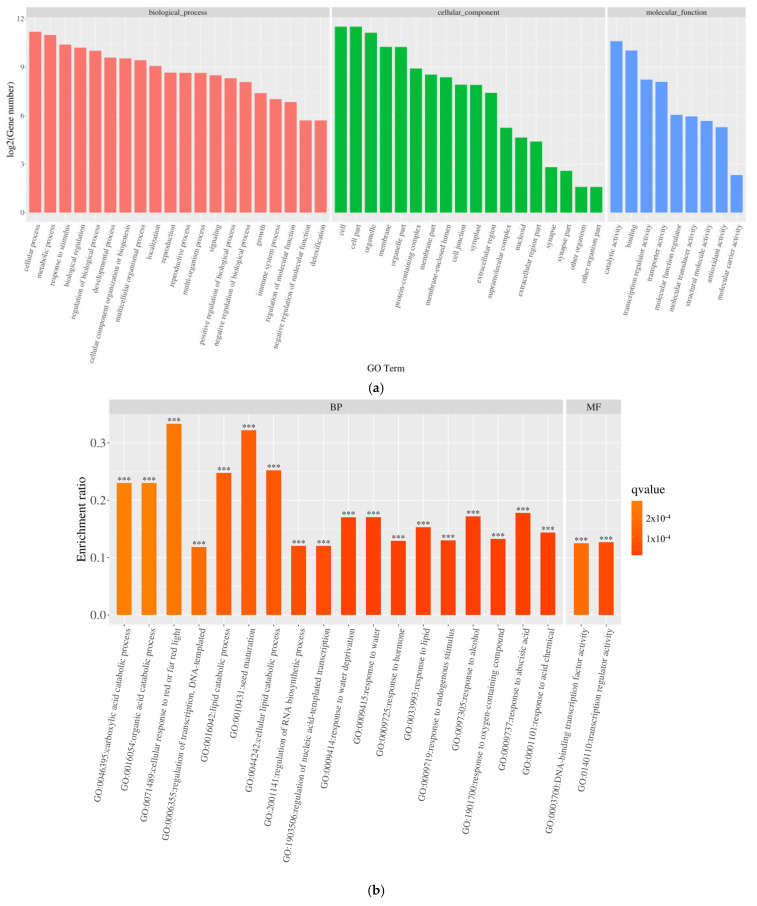

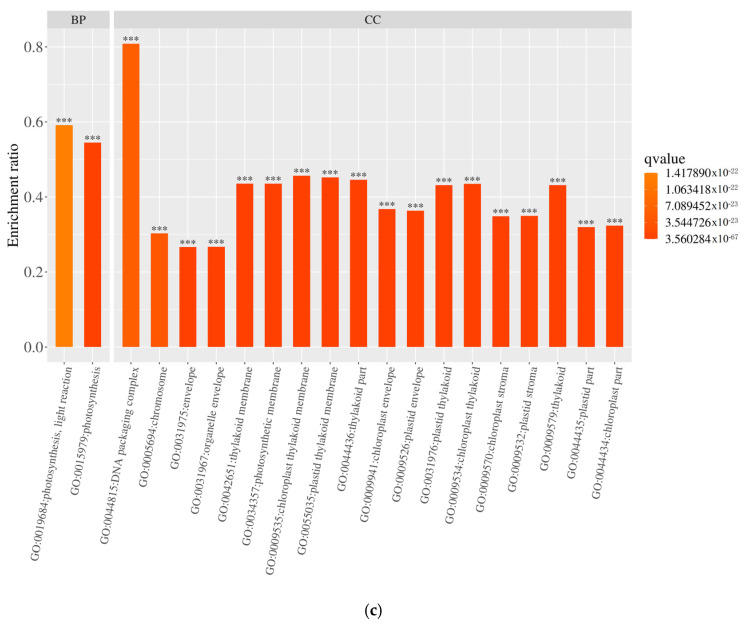
Gene ontology (GO) classification of differentially expressed genes (DEGs): (**a**) functional distribution across biological processes (BP), cellular components (CC), and molecular functions (MF); (**b**) GO enrichment of upregulated DEGs; (**c**) GO enrichment of downregulated DEGs. ***: *p* < 0.001.

To delve into the potential biochemical processes associated with the differentially expressed genes, the 6219 genes underwent GO classification analysis, spanning categories such as biological process (BP), cellular component (CC), and molecular function (MF). As depicted in Figure 6a, compared to the control group, the differentially expressed genes under salt stress were notably distributed across subcategories within biological processes, cellular components, and molecular functions. Notably, biological processes were concentrated in subcategories like “cellular processes,” “metabolic processes,” “single organism processes,” “biological regulation and biological process regulation,” and “response to stimulation.” Cellular components primarily encompassed subcategories such as “cells,” “cell parts,” “cell organs,” and “membranes,” while molecular functions were chiefly concentrated in subcategories like “catalytic activity,” “transcriptional regulatory activity,” “transporter active molecules,” “molecular transduction activity,” and “structural molecular activity.”

The GO enrichment analysis of DEGs in pea sprouts subjected to salt stress is depicted in Figure 6b,c. The outcomes reveal that the enrichment of upregulated DEGs primarily focuses on biological processes, notably “response to acidic substances,” succeeded by pathways such as “abscisic acid response,” “cell response to red light or far-red light,” “transcriptional regulation, DNA template,” “lipid decomposition metabolic process,” and “seed maturity.” Conversely, downregulated DEGs are predominantly enriched in the realm of photosynthesis, particularly “photosynthesis, light response.” Within cellular components, no pathways exhibited significantly enriched upregulated DEGs, whereas downregulated DEGs were notably enriched in categories like “chloroplast part,” “chromosome,” “capsule,” “organelle envelope,” and “chloroplast part,” among others. Notably, pathways with significantly enriched upregulated DEGs, such as “DNA-binding transcription factor activity” and “transcriptional regulation activity”, experienced substantial increases within the molecular function category, underscoring the pivotal role of these pathways in the response of pea sprouts to salt stress.

**Figure 7:**
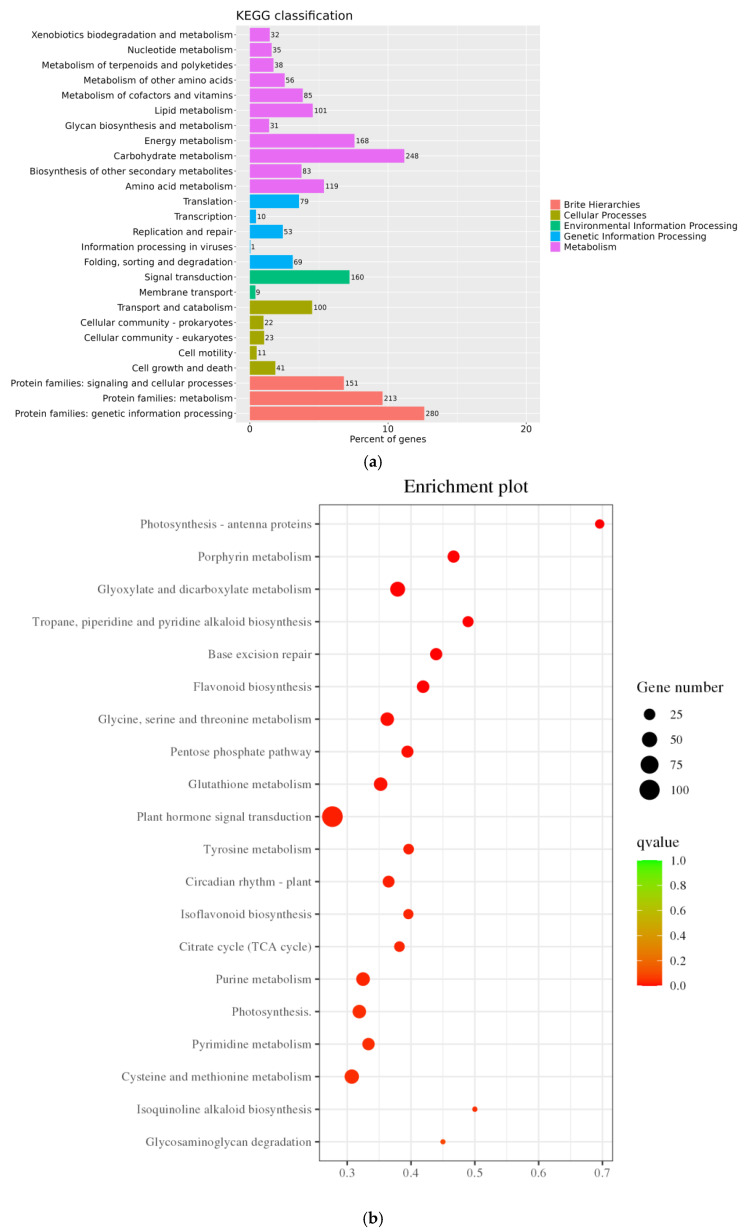

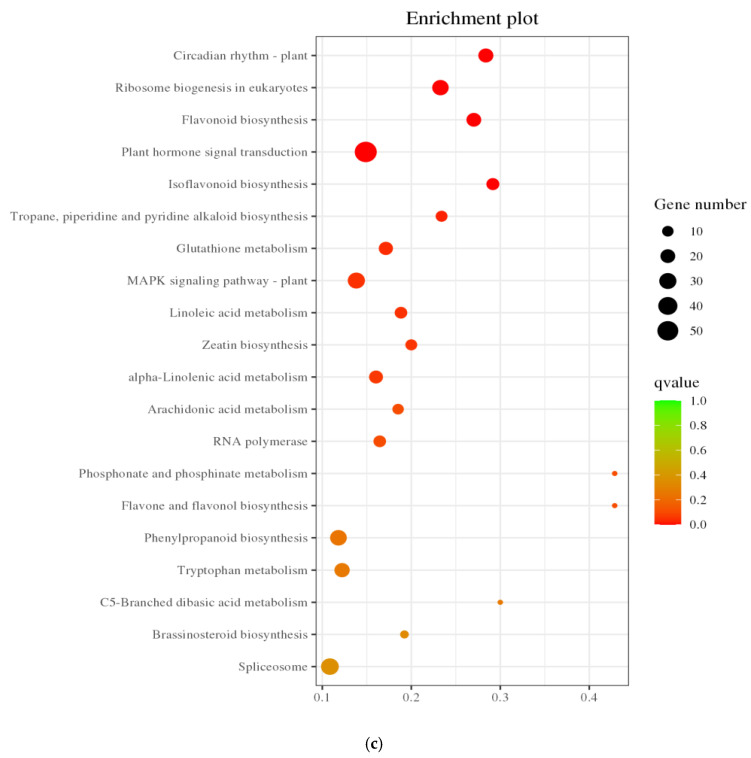

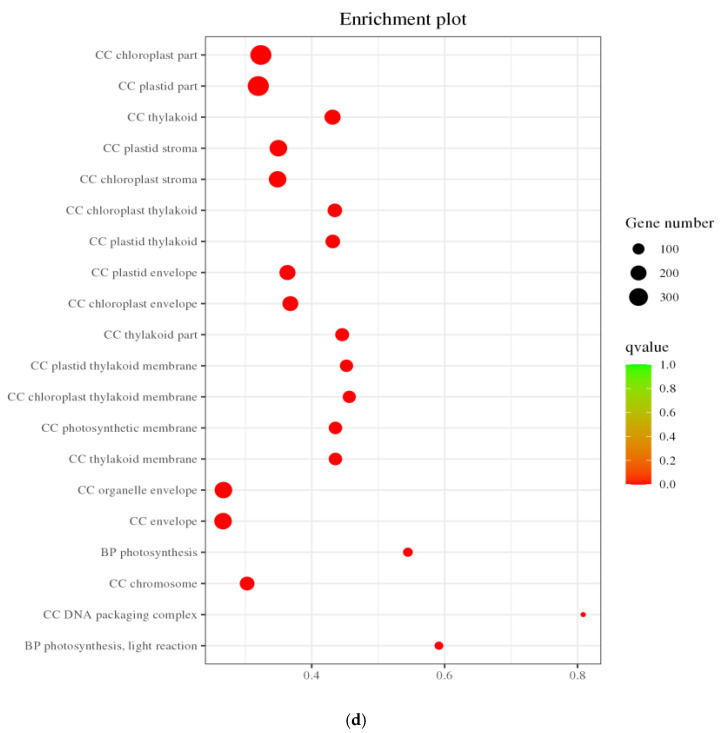
KEGG annotation classification statistics plot (**a**); Bubble plots of all DEGs and KEGG enrichment pathways (**b**); Bubble chart of upregulated DEGs KEGG enrichment pathway (**c**); and Downregulated DEGs KEGG enrichment pathway (**d**).

Through KEGG annotation and the visualization of functional distribution maps, significant alterations across 1622 pathways were observed. As illustrated in Figure 7a, DEGs were notably enriched in pathways like “Photosynthesis–Antenna Protein,” “Porphyrin Metabolism,” “Glyoxylate and Dicarboxylic Acid Metabolism,” “Alkaloid Biosynthesis,” and “Base Excision Repair,” with “Plant Hormone Signal Transduction” emerging as the pathway harboring the largest number of DEGs. In Figure 7b,c, under salt treatment conditions, upregulated DEGs were predominantly enriched in “Circadian Rhythm Plant,” followed by pathways like “Biogenesis of Ribosomes in Eukaryotes,” “Flavone Biosynthesis,” and “Plant Hormone Signal Transduction,” with “Plant Hormone Signal Transduction” and “MAPK Signaling Pathway Plant” standing out as the pathways with the highest DEG counts. Conversely, KEGG pathways enriched with downregulated DEGs were primarily associated with “Photosynthesis–Antenna Protein,” “Glyoxylate and Dicarboxylic Acid Metabolism,” “Photosynthesis,” “Porphyrin Metabolism,” and “Glycine, Serine, and Threonine Metabolism,” with “Photosynthesis” housing the most DEGs. Notably, within the photosynthesis-related DEGs, a single DEG was upregulated while 36 were downregulated, underscoring the pronounced impact of salt stress on the photosynthesis pathway in pea sprouts. These pathways potentially play crucial roles in the response of pea sprouts to salt stress, although some pathways exhibited enrichment specific to certain tissues and time points.

### 3.5. Metabolic Pathways of Transcriptional Response in Pea Sprouts Under Salt Stress

**Figure 8:**
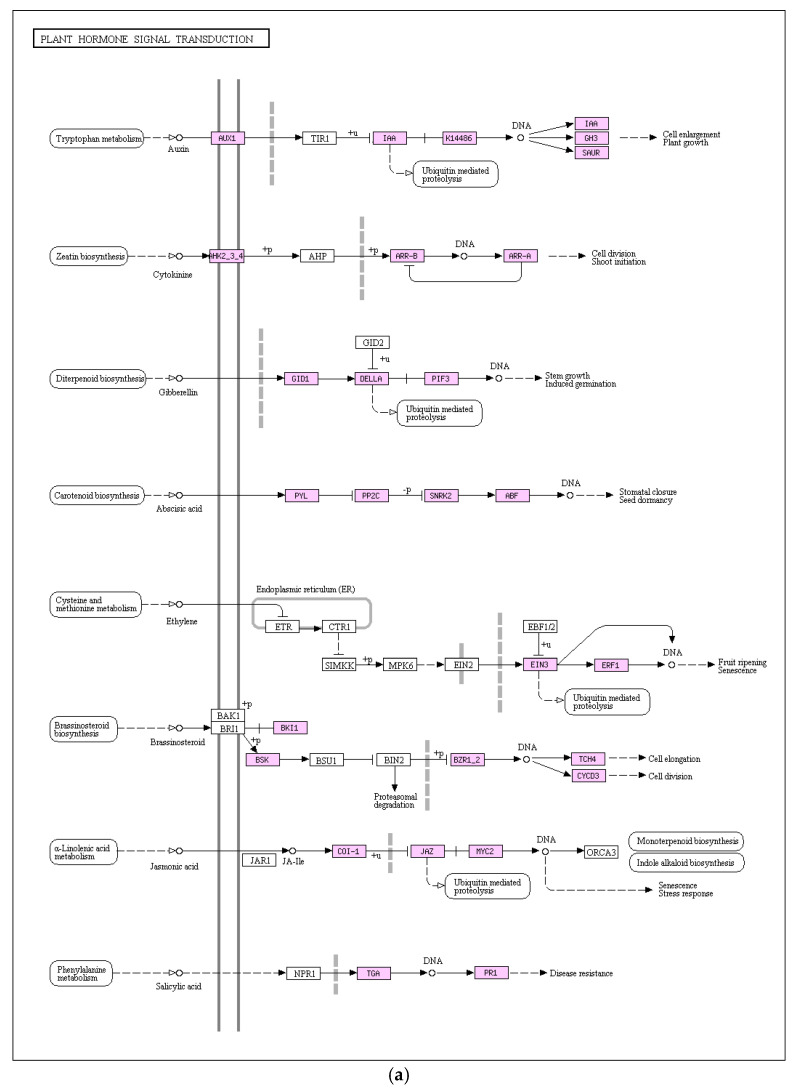

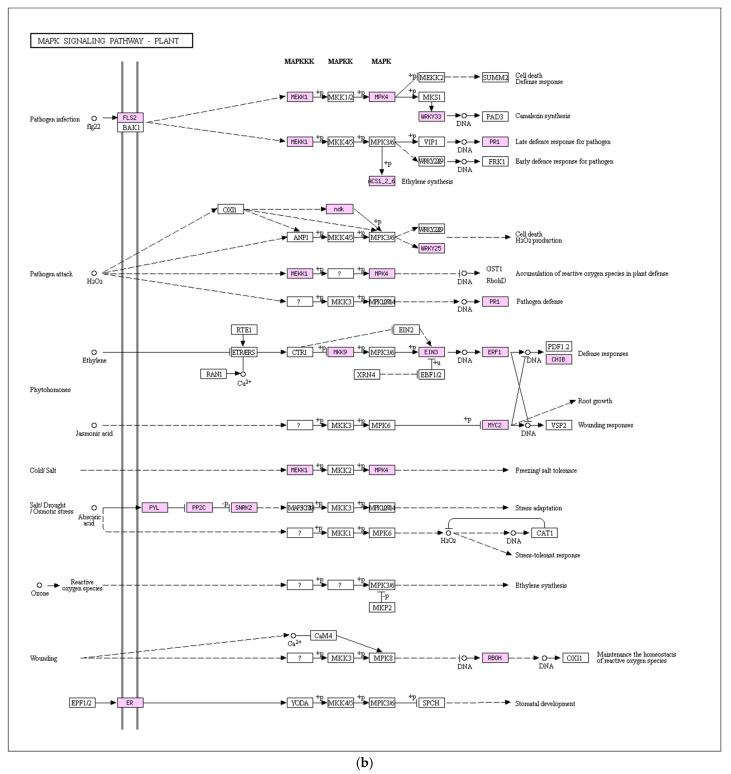
Key enriched signaling pathways: (**a**) Plant hormone signal transduction with 106 DEGs (57 upregulated, 49 downregulated); (**b**) Mitogen-activated protein kinase (MAPK) signaling pathway with 47 DEGs (30 upregulated, 17 downregulated).

Plant hormones serve as vital physiological regulators, influencing various physiological processes at remarkably low concentrations and playing a pivotal role in acclimating to challenging environmental conditions. The KEGG enrichment analysis results displayed in Figure 8a revealed that plant hormone signal transduction stood out as a significant pathway with enriched DEGs. In comparison to the control group, the salt-stressed pea sprouts exhibited a total of 106 DEGs within this pathway (57 upregulated and 49 downregulated). Studies have found that when plants respond to abiotic stress, a variety of hormone-mediated molecular mechanisms will occur. Examination of gene expression alterations, including ABA, JA, and IAA, revealed that salt stress incites a diverse array of hormone responses in pea sprouts. Notably, the PYL protein family, pivotal in the ABA signal transduction pathway, demonstrated significant downregulation in pea sprouts under salt stress, while 8 PP2C genes exhibited upregulation, hinting at the potential importance of the interaction between PYL and PP2C genes in the salt stress response of pea sprouts. MYC2, a transcription factor crucial for JA response, exhibited one upregulated gene. Within the auxin signal transduction pathway, Aux/IAA genes, crucial inducers, displayed regulatory effects on plant response to auxin, with 13 IAA genes identified, among which 3 were upregulated and 10 downregulated. Hormone signal transduction emerges as a critical player in the salt stress response of pea sprouts.

The MAPK signal transduction pathway stands as an important component of plant growth and development, aiding plants in effectively combating external environmental stressors, including pathogen invasions. As depicted in Figure 8b, transcriptome analysis outcomes highlight the involvement of genes linked to this pathway in the response of pea sprouts to salt stress, showcasing significant differential expressions. In comparison to the control group, the salt-stressed pea sprouts exhibited 30 upregulated DEGs and 17 downregulated DEGs within the MAPK signaling pathway. The PP2C gene family exerts an inhibitory effect on the MAPK signaling pathway, with eight upregulated PP2C gene expressions identified under salt stress. Thus, the plant’s feedback mechanism within the MAPK signaling pathway plays a pivotal role in enhancing salt stress resistance in pea sprouts.

## 4. Discussion

### 4.1. ABA-Centric Coordination of Early Antioxidant Defense

Salt stress imposes a multifaceted challenge on pea sprouts, eliciting a tightly coordinated response that spans physiological, biochemical, and transcriptomic levels. At the heart of this adaptive strategy lies abscisic acid (ABA), which orchestrates a phased cascade of events, integrating early antioxidant defenses, metabolic reconfiguration, and gene expression changes into a cohesive framework for survival.

The onset of salt stress rapidly triggers the accumulation of reactive oxygen species (ROS), which serves as a signal to activate ABA biosynthesis within hours, as evidenced by hormonal profiling (Figure 2). This ABA surge acts as a pivotal regulator, driving the biphasic response of the ascorbate–glutathione (AsA-GSH) cycle, a critical antioxidant system (Figure 1). Mechanistically, ABA enhances the transcription of genes such as *Ascorbate peroxidase* (*APX*) by interacting with ABA-responsive elements (ABREs) in their promoter regions, a process well-documented in plant stress responses [22]. This molecular interaction accounts for the observed transient spike in APX activity during the early phase of stress (Figure 1a), effectively neutralizing ROS-induced oxidative damage. Simultaneously, ABA upregulates *Glutathione reductase* (*GR*) expression, promoting the accumulation of glutathione (GSH) despite a notable depletion of its precursor, serine (Figure 1e and Figure 3) [8]. This GSH buildup establishes a positive feedback loop by stabilizing ABA biosynthetic enzymes, such as *Aldehyde oxidase* (AO_3_), thereby amplifying ABA signaling [21]. Far from functioning as an isolated mechanism, the AsA-GSH cycle emerges as a dynamic, ABA-integrated redox network that not only mitigates oxidative stress but also amplifies the plant’s stress signaling capacity.

### 4.2. Metabolic Reconfiguration Fueled by Hormonal Trade-Offs

As salt stress intensifies, hormonal dynamics shift dramatically, redirecting metabolic resources from growth to defense (Figure 2). A striking decline in indole acetic acid (IAA) levels suppresses sucrose synthase activity, leading to reduced sucrose availability and diminished pools of Calvin cycle intermediates like sedoheptulose-7-phosphate (S7P) and ribulose-1,5-bisphosphate (RuBP) (Figure 4) [31]. In contrast, the concurrent rise in ABA levels reallocates carbon toward osmoprotective functions. Despite the transcriptional upregulation of serine biosynthetic genes such as *Phosphoglycerate dehydrogenase* (*PGDH*; KEGG pathway ko00260), serine levels drop (Figure 3) because it is preferentially channeled into GSH synthesis to bolster antioxidant defenses. Additionally, the reduction in organic acids like phosphoglyceric acid (PGA) and phosphoenolpyruvate (PEP) reflects ABA’s inhibition of phosphoenolpyruvate carboxylase, which diverts carbon flux away from the TCA cycle and toward the production of osmotic solutes [32]. This metabolic trade-off, sacrificing growth-related metabolites for defense compounds, is energetically sustained by jasmonic acid (JA). The elevation of JA (Figure 2) supports mitochondrial function and ATP production under stress, providing the energy needed for GSH synthesis and other defense processes [28]. Together, these hormonal shifts illustrate a strategic reorientation of metabolic priorities, seamlessly linked to ABA’s overarching regulatory influence.

### 4.3. Transcriptional Consolidation of Physiological Adaptations

The transcriptomic response to salt stress builds upon these early biochemical adjustments, institutionalizing them into a stable, long-term adaptation strategy (Figure 6, Figure 7 and Figure 8). A prominent feature is the downregulation of photosynthesis-related genes (Figure 7d), which codifies ABA-induced growth arrest to conserve resources. The presence of ABRE motifs in these genes confirms ABA’s direct regulatory role [33]. Meanwhile, the upregulation of *PP2C* genes sustains ABA sensitivity by modulating phosphatase activity, ensuring that the stress signal remains robust (Figure 8a). This is complemented by the induction of MAPK signaling pathways, priming the plant for potential secondary stresses (Figure 8b). As the stress response matures, the downregulation of *Monodehydroascorbate reductase* (*MDHAR*) and *Dehydroascorbate reductase* (*DHAR*) aligns with suppressed glutathione metabolism genes (KEGG ko00480), reflecting a reduced demand for antioxidant activity once GSH levels stabilize (Figure 1b,c). Notably, 78% of differentially expressed genes (DEGs) in enriched pathways, such as “Plant hormone signal transduction,” contain ABRE motifs, underscoring ABA’s position as the genomic architect of salinity tolerance [33]. This transcriptional reprogramming effectively locks in the physiological and biochemical changes initiated earlier, ensuring a durable adaptive state.

The response to salt stress unfolds as a self-reinforcing cascade: an initial ROS burst triggers ABA accumulation, which simultaneously activates the AsA-GSH cycle, suppresses IAA while elevating JA, reallocates metabolic resources, and drives ABRE-mediated transcriptomic reprogramming. This model accounts for the observed synergies—early ABA elevation boosts antioxidant defenses (e.g., APX surge; Figure 1a) while repressing growth-related processes (e.g., sucrose decline; Figure 4). The transcriptome then reinforces these shifts, securing sustained tolerance. Our results capture these dynamic transitions, providing a mechanistic clarity absent in previous studies of pea under salt stress [2]. By integrating these phases, we reveal a holistic adaptive strategy where each component builds logically upon the last. Nonetheless, further exploration is warranted to unveil potential tissue-specific responses within the same biological process and to scrutinize the temporal dynamics of differentially expressed genes [34].

## 5. Conclusions

This study elucidates the multifaceted physiological, biochemical, and transcriptomic responses of pea sprouts to salinity stress, revealing a dynamic interplay of antioxidant defense systems, hormonal reconfiguration, metabolic adjustments, and gene regulatory networks. Our findings demonstrate that pea sprouts employ a biphasic strategy in their antioxidant response, characterized by an initial surge in ascorbate peroxidase (APX) activity and ascorbic acid (AsA) content, followed by a gradual decline under prolonged salt exposure. This transient enhancement of the ascorbate–glutathione (AsA-GSH) cycle highlights the plant’s capacity to temporarily mitigate oxidative damage, though sustained stress ultimately overwhelms its antioxidant defenses. Metabolomic perturbations, particularly in sugars, amino acids, and organic acids, reflect a metabolic shift toward osmotic adjustment and energy conservation, while transcriptomic profiling identifies 6219 differentially expressed genes (DEGs) enriched in photosynthesis, hormone signaling, and stress-responsive pathways. Notably, the downregulation of photosynthesis-related genes and upregulation of MAPK and ABA signaling components suggest that pea sprouts prioritize stress survival over growth under high salinity. These insights not only deepen our understanding of pea sprout resilience but also provide a molecular framework for improving salt tolerance in leguminous crops.

## Data Availability

Data are contained within the article.
